# Glioblastoma Multiforme Masquerading as Stroke

**DOI:** 10.7759/cureus.15230

**Published:** 2021-05-25

**Authors:** William Remley, Nitin Butala

**Affiliations:** 1 Neurology, Lake Erie College of Osteopathic Medicine, Jacksonville, USA; 2 Neurology, Baptist Medical Center Jacksonville, Jacksonville, USA

**Keywords:** glioma, glioblastoma, multiforme, temporal, tumor, anomic, aphasia, eeg

## Abstract

Sudden-onset anomic aphasia is a unique symptom that is suggestive of an acute etiology. This case presents a sudden-onset focal neurological deficit with an underlying brain tumor. A 68-year-old female awoke with sudden-onset anomic aphasia, with mild hypertension as her only medical history. After an initial stroke workup was unremarkable, magnetic resonance imaging found a focal lesion on the left temporal lobe. An electroencephalogram showed lateralized periodic discharge and a focal area of increased epileptic potential in the left temporal lobe. Brain biopsy revealed World Health Organization grade IV glioblastoma, followed by resection. This case is an important reminder that chronic etiologies may present with acute symptoms.

## Introduction

In an effort to not miss stroke diagnoses, many etiologies fall into a group known as stroke mimics. Stroke mimics are etiologies that present with symptoms similar to a stroke but result in a different diagnosis. The identification of stroke mimics is critical to reducing unnecessary risks that arise with the progressing landscape of interventions for acute stroke, such as mechanical thrombectomy or chemical thrombolysis. Of all patients with suspected stroke, 15-19% are diagnosed with a stroke mimic [1,2]. The most common etiology of a stroke mimic is a seizure, responsible for 19-28% of stroke mimics [1,3]. Stroke mimics contrast from stroke chameleons, which are strokes that mimic another diagnosis. Clinicians must consider these two groups when determining which patients may benefit from reperfusion therapies [4].

In addition to a patient’s presenting symptoms, the timing of the symptoms also contributes to the differential diagnosis. Sudden-to-acute-onset symptoms are typically associated with ischemic events, seizures, and toxic and metabolic causes, while chronic-onset symptoms are typically associated with brain tumors, degenerative diseases, or inflammatory processes [5]. Here, we present a unique case of sudden-onset anomic aphasia, or word-finding difficulty, presenting with a glioblastoma multiforme (GBM).

## Case presentation

A 68-year-old, right-handed Caucasian female presented with sudden-onset word-finding difficulty, speech hesitation, and difficulty following commands upon awakening from a nap. No other neurological deficits were observed. Her only medical history was a recent diagnosis of mild hypertension two weeks prior. For this, she was started on and had inconsistently been taking amlodipine 5 mg. Her initial vital signs showed that she was afebrile (99.6°F) with a heart rate of 109 beats/minute and blood pressure of 129/94 mmHg. Physical examination revealed a National Institutes of Health Stroke Scale score of 4, which consisted of one point for answering the month incorrectly, one point for performing one task incorrectly when instructed to open and close eyes and then grip and release her hand, and two points for severe aphasia.

Basic metabolic panel showed sodium 133 mEq/L, potassium 3.6 mEq/L, chloride 99 mEq/L, CO_2_ 21 mEq/L, glucose 234 mg/dL, blood urea nitrogen 18 mg/dL, creatinine 0.87 mg/dL, and calcium 8.7 mg/dL. Complete blood count showed white blood cell (WBC) 12.01 K/mcL, red blood cell (RBC) 4.29 mil/mcL, hemoglobin 14.0 g/dL, hematocrit 41.8%, and platelets 297 K/mcL.

**Figure 1:**
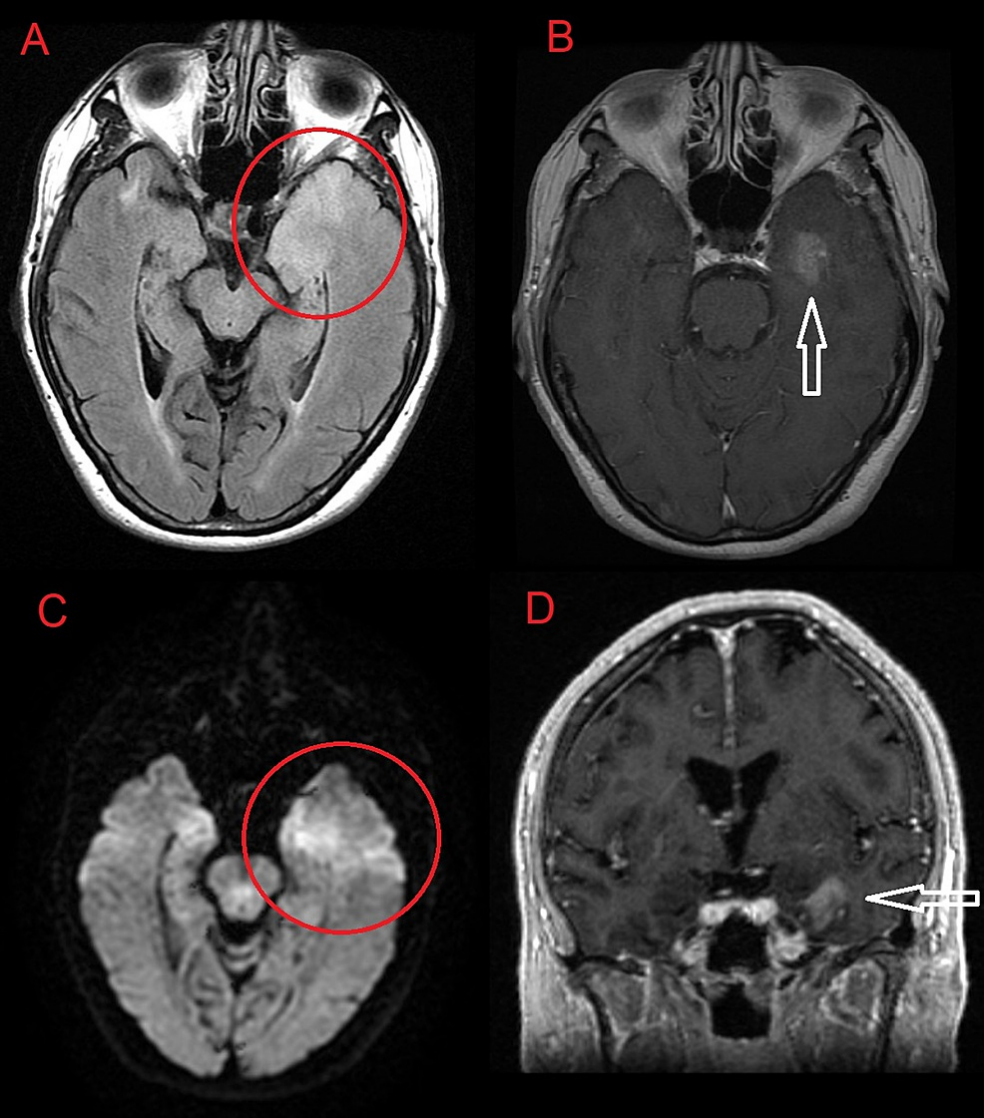
MRI of the brain. Axial FLAIR (A), T1-weighted with contrast (B), diffusion-weighted (C), and coronal T1-weighted with contrast (D) imaging of the brain demonstrate a 1.8 × 1.6 cm area of cortical and subcortical FLAIR hyperintensity (A) with a small internal focus of mild diffusion restriction (C) and amorphous enhancement in the anteromedial aspect of the left temporal lobe involving the uncus, amygdala, and hippocampal head (B, D). FLAIR: fluid-attenuated inversion recovery; MRI: magnetic resonance imaging

Computed tomography (CT) without contrast revealed an area of hypoattenuation in the deep and subcortical white matter of the right parietal lobe, which could reflect an evolving infarct although nonspecific. Subsequent CT angiography and CT perfusion could not identify vascular territory ischemia or infarct. Because the patient was last known well more than 4.5 hours ago and based on imaging studies, she was not a candidate for receiving tissue plasminogen activator. Sixteen hours later, she still had hesitation producing words, although her word output had significantly improved. Magnetic resonance imaging (MRI) with and without contrast were performed. MRI of the brain without contrast showed a 5.1 × 2.6 cm area of hyperintensity in the left temporal lobe, while MRI with contrast showed a 1.8 × 1.6 cm area of hyperintensity in the left temporal lobe (Figure 1).

**Figure 2:**
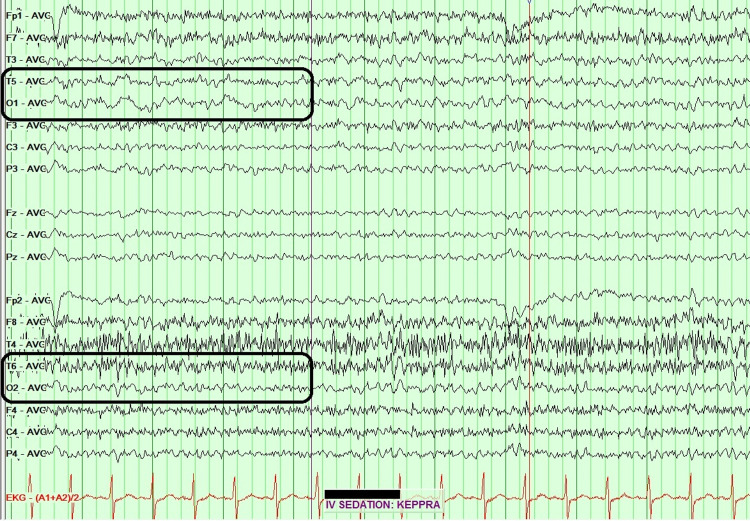
EEG reviewed with average montage. More pronounced slowing is seen over the left posterior temporal and occipital region (T5 and O1 electrodes), in the 2-3 Hz range, compared to a normal range of 12-13 Hz in the right posterior temporal and occipital region (T6 and O2 electrodes). EEG: electroencephalogram

The MRI findings were concerning for several etiologies, narrowing the differential diagnoses to infectious encephalitis, autoimmune limbic encephalitis, neoplasm, and postictal edema (Figure 1). Acute herpes encephalitis had to be considered and was clinically excluded, although there was no involvement of other limbic structures, such as insula or cingulate gyrus, no hemorrhage, and only a small volume of mild diffusion restriction. Diffusion restriction smaller than the area of fluid-attenuated inversion recovery (FLAIR) signal abnormality made a recent infarct unlikely (Figures 1A, 1C). Autoimmune/limbic encephalitis could have had this appearance but is often bilateral making this unlikely. Postictal edema was less likely given the degree of mass effect and the lack of involvement of additional limbic structures such as the hippocampal body/tail or cingulate gyrus. With the given differential, the patient was started on empiric acyclovir and levetiracetam, and a lumbar puncture was performed. A lumbar puncture and cerebrospinal fluid study revealed clear fluid, WBCs 6/mm³, RBCs 3/mm³, 70% neutrophils, 25% lymphocytes, glucose 77 mg/dL, protein 32 mg/dL, cryptococcal Ag negative, and non-reactive Venereal Disease Research Laboratory test. Herpes simplex virus polymerase chain reaction was negative. Hence, acyclovir was discontinued. Electroencephalogram (EEG) showed lateralized periodic discharge in the left frontotemporal region with a focal region of increased epileptic potential likely from the underlying structural abnormality (Figure 2).

A brain biopsy was needed to further identify the FLAIR hyperintensity seen on MRI. The brain biopsy and resection revealed glioblastoma, isocitrate dehydrogenase-wildtype, World Health Organization grade IV with an O6-methylguanine-DNA-methyltransferase gene promoter methylation of 75.76%. At discharge, the patient was clinically doing well. Her treatment plan consisted of chemotherapy and radiation.

## Discussion

While the differential diagnosis in patients presenting with an acute focal neurological deficit requires that an acute ischemic etiology be explored first and ruled out due to time-sensitive interventions such as mechanical thrombectomy and chemical thrombolysis, clinicians must also consider other diagnoses that may also describe the sudden-onset symptoms, such as seizures induced by a cranial neoplasm. In our case, the patient was found to have a glioblastoma as the underlying etiology. Grade IV glioblastoma can also be referred to as a grade IV astrocytoma or GBM. GBM is the most commonly occurring brain tumor [6]. It has an incidence of 3.2 per 100,000 adults per year and accounts for 14% of all primary brain and other central nervous system tumors [6]. GBM can present with symptoms such as headache, visual disturbances, seizures, or focal neurological deficit. A 2018 meta-analysis analyzing the most common presenting symptoms due to gliomas established that aphasia was the seventh most common symptom, with other more common symptoms being seizures (37%), cognitive deficits (36%), drowsiness (35%), dysphagia (30%), headache (27%), confusion (27%), aphasia (24%), motor deficits (21%), fatigue (20%), and dyspnea (20%) [7]. Anomic aphasia is a unique symptom that has been shown to be associated with damage to the left temporal lobe [7-9]. A 2018 study examining patients with lesions in the left temporal lobe suggested that damage to this area results in difficulty in lexical selection [10]. Of the fluent aphasias, Wernicke’s aphasia occurs most commonly. Both anomic aphasia and Wernicke’s aphasia are fluent aphasias, but Wernicke’s aphasia can be distinguished by its markedly impaired comprehension, repetition, and an inability to read and write. Classically, Wernicke’s aphasia presents as a voluminous “word salad” that is characteristically meaningless and associated with impaired comprehension.

To our knowledge, there has been only one other published case report of anomic aphasia due to a left temporal lobe glioblastoma. A 2013 case report by Feng et al. described a patient with an onset of anomic aphasia over a period of one month [11]. The case report by Feng et al. aligns with a more indolent development of aphasia compared to our patient’s sudden-onset aphasia. Although the two cases display similar symptoms, their contrasting time courses suggest different mechanisms of aphasia. Sudden-onset aphasia suggests an acute pathology such as seizure-induced aphasia which contrasts with a chronic development of symptoms which suggests a chronic pathology such as mass effect.

## Conclusions

Although previous reports have correlated damage of the left temporal lobe with word-finding difficulty, this is the first reported case of sudden-onset anomic aphasia due to a left temporal lobe GBM. The uniqueness of this case comes from the patient’s acute onset of symptoms, suggesting seizure. This case is an example of how appropriate clinical reasoning is critical in reaching the diagnosis of unusual presentations. Although the neoplasm was a chronic process, this case is a reminder that acute symptoms can result from chronic etiologies.
